# *Perp* Deficiency Induces Defective Negative Selection and Autoimmune Arthritis in Aged Mice

**DOI:** 10.1111/acel.70514

**Published:** 2026-04-23

**Authors:** Yan Zhou, Junrong Li, Xiao Leng, Jiao Shi, Gan Zhang, Shan Chen, Dong Liu, Qian Deng, Yan He, Guixiu Shi, Ying Xu, Yuan Liu, Yantang Wang

**Affiliations:** ^1^ Department of Emergency West China Second University Hospital and Key Laboratory of Birth Defects and Related Diseases of Women and Children (Ministry of Education), Sichuan University Chengdu China; ^2^ Clinical Laboratory, Clinical Medical College and the First Affiliated Hospital of Chengdu Medical College Chengdu Medical College Chengdu China; ^3^ Department of Immunology, School of Basic Medical Sciences Chengdu Medical College Chengdu China; ^4^ The Second Affiliated Hospital of Chengdu Medical College China National Nuclear Corporation 416 Hospital Chengdu China; ^5^ Department of Pharmacology, School of Pharmacy Chengdu Medical College Chengdu China; ^6^ Department of Rheumatology and Clinical Immunology The First Affiliated Hospital of Xiamen University, School of Medicine, Xiamen University Xiamen China

**Keywords:** aging, apoptosis, autoimmune arthritis, negative selection, *Perp*, T‐cell

## Abstract

Thymic negative selection is characterized by the apoptosis of autoreactive thymocytes and plays a critical role in maintaining self‐tolerance. Numerous apoptosis‐related genes influence cell fate during T‐cell development. The PERP protein functions in apoptosis induction and as a tumor suppressor; however, p53 targets the *Perp* promoter, leading to its downregulation in various cancers. We investigated the specific role of *Perp* by studying conditional knock‐out mice exhibiting partial thymic T‐cell development defects and a significant accumulation of thymic CD4SP T‐cells. Ex vivo and in vivo analyses revealed that *Perp* regulates the survival of thymic T‐cell subsets during clonal deletion, particularly CD4SP T‐cells following TCR stimulation. These floxed mice also exhibited an expansion of the Helios^+^ CD4SP thymocyte population. Moreover, middle‐aged floxed mice exhibited excessive accumulation of activated CD4^+^ T‐cells in peripheral blood, alongside T cell‐mediated autoimmune arthritis. These findings indicate that conditional *Perp* knockout mice exhibit a deficiency in thymic negative selection and heightened susceptibility to autoimmunity with aging.

## Graphical abstract

*Perp* deficiency promotes age‐related autoimmunity.

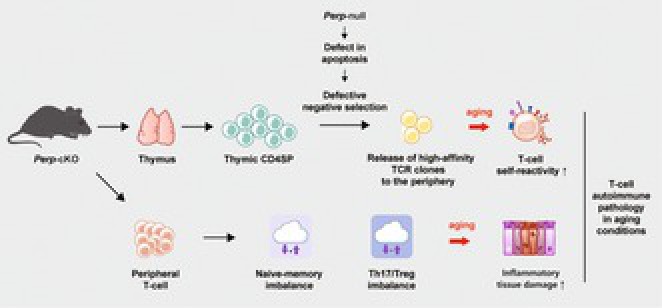

## 1 Introduction

Thymic negative selection is not a single event but a progressive screening process. The cortex is the site of the primary wave of deletion, accounting for roughly 78% of clonal elimination at the double positive (DP) stage. This wave is focused on high‐throughput removal of ubiquitous self‐reactivity (Ashby and Hogquist 2023). The medulla represents the secondary wave of selection, handling the remaining 22% of deletion events. Although lower in volume, its complexity is vastly superior, utilizing the AIRE‐regulated TCR repertoire to ensure tolerance to peripheral tissues by mTECs (Breed et al. 2019). The elimination of self‐reactive clones is primarily distributed in the thymic cortex in terms of absolute cell numbers, but the medulla remains the primary site for the functional diversification and fine‐tuning that prevents organ‐specific autoimmunity. There is evidence that several apoptosis‐related genes function in the process of thymic negative selection (Opferman 2008). For example, BCL2 like 11 protein (also known as BIM), which contains a Bcl‐2 homology domain 3 (BH3), promotes the mitochondrial (intrinsic) apoptosis cascade. The level of BIM correlates with signaling through TCRs, and *Bim* knock‐out mice have defective thymic negative selection (Erlacher et al. 2006). The loss of BAX and BAK also leads to T lymphocyte abnormalities, and DP T‐cells in *Bak* ^*‐/‐*^ *Bax* ^*‐/‐*^ reconstituted mice do not undergo negative selection due to a defect in apoptosis (Rathmell et al. 2002). Strong signaling via TCRs results in negative selection and also induces NUR77, a nuclear receptor that functions in apoptosis and is implicated in the process of negative selection (Mittelstadt et al. 2019). Further studies of proteins that function in the apoptosis of thymocytes may therefore provide insights into the development of T‐cell tolerance and the development of autoimmunity (Deng et al. 2025).

The p53 apoptosis effector related to PMP22 (PERP) is a tetraspan protein located in the plasma membrane whose transcription is regulated by p53 and p63 (Dusek et al. 2012). Ihrie et al. (2005) first reported that the *Perp* gene was targeted by p63, and that the PERP protein functioned in epithelial integrity. More recent studies suggested that *PERP* functioned in apoptosis, was targeted by p53/p63, and was a tumor suppressor that was downregulated in several human cancers (Holmes et al. 2020). More specifically, downregulation of *PERP* by tumor cells leads to evasion of apoptosis, and often occurs early during tumorigenesis (Roberts and Paraoan 2020). *PERP* is expressed in many tissues and in the organs of the immune system (Beaudry et al. 2010; Ihrie et al. 2005). We previously studied floxed mice that had a conditional knock‐out (Lck‐Cre × *Perp* ^fl/fl^), and demonstrated that loss of *Perp* in their T‐cells increased the resistance to anti‐Fas‐induced apoptosis in their Th17 cells. We also found that this response was accompanied by inhibition of the caspase‐dependent intrinsic pathway of apoptosis, promotion of experimental autoimmune encephalomyelitis (EAE), and increased levels of inflammation and demyelination in the central nervous system (Zhou et al. 2018). Moreover, other research showed that peripheral *Perp* ^−/−^ CD4^+^ effector memory T‐cells predominated over wild‐type cells, leading to enhanced cellular resistance to apoptosis in adoptive transfer assays and lymphopenia‐induced proliferation (Zhou et al. 2020). Due to its important role in regulating the survival of immune cells, *Perp* appears to affect the development and function of T‐cells at multiple levels.

The p53 pathway is deeply intertwined with the age‐related remodeling of the immune system. This includes both the failure of the immune system to clear senescent cells and the inherent senescence of the immune cells themselves (Tizazu et al. 2025). Terminally differentiated memory T cells—which accumulate in the elderly—are often resistant to apoptosis and show characteristics of highly inflammatory, cytotoxic, but proliferation‐incompetent effectors (Lorenzo et al. 2022). A decreased level of *Perp* increases the survival of CD4^+^ effector T cells and memory phenotypes, and there is therefore likely to be a negative correlation between the level of *Perp* and tolerance of peripheral T cells. Moreover, because peripheral mature CD4^+^ T cells from the thymus develop due to clonal deletion, we hypothesize that a decreased level of *Perp* might facilitate the generation of defective CD4^+^ T cells by disruption of thymic negative selection and result in increased risks for autoimmune disease, particularly under aging conditions. To test this hypothesis, we first crossed Lck‐Cre transgenic (Tg) mice with *Perp* ^fl/fl^ mice to generate Lck‐Cre‐specific *Perp* conditional knock‐out mice (Lck‐Cre × *Perp* ^fl/fl^). Then, we crossed these Lck‐Cre × *Perp* ^fl/fl^ mice with 3A9 or OT‐II TCR Tg mice to allow direct tracing of clonotype‐specific T cells after binding to cognate antigens. Our general purpose was to examine the role of *Perp* in regulating autoreactive thymocytes during thymic negative selection and the possible role of this process in autoimmune diseases.

## 2 Materials and Methods

### 2.1 Mice

*Perp* ^fl/fl^ mice (strain No. 002597) and 3A9 TCR Tg mice (strain No. 016132) were obtained from Jackson Laboratories (ME, USA). Lck‐Cre mice (strain No. J003802) were obtained from Model Animal Research Center of Nanjing University. OT‐II TCR Tg mice and RIP‐mOVA Tg mice were the kind gift of Professor Guixiu Shi (Xiamen University, Xiamen, China). Conditional knockout mice (Lck‐Cre × *Perp* ^fl/fl^) in C57BL/6 background were generated as described previously (Zhou et al. 2018). All genetically engineered mice had the C57BL/6 background and were maintained in a specific pathogen‐free environment at Chengdu Medical College in accordance with the China Regulations for the Administration of Affairs Concerning Experimental Animals. All animal protocols were approved by the Animal Care and Use Committee of Chengdu Medical College (Permission No. CMC‐IACUC‐2018030801). The sample sizes are indicated in the figure legends or figures. Mice were allocated at random to experimental groups.

### 2.2 Bone Marrow Chimeras

For RIP‐OVA chimeras, wild type and RIP‐mOVA recipient mice were lethally irradiated with X‐rays (9.5 Gy) using an X‐RAD 225 small animal irradiator (Precision X‐Ray). The Lck‐Cre × *Perp* ^fl/fl^ mice were crossed with mice Tg for the OT‐II TCR that was specific for a peptide corresponding to residues 323 to 339 of chicken ovalbumin (OVA) presented by H‐2^b^. Bone marrow cells from OT‐II; Lck‐Cre × *Perp* ^fl/+^ mice or OT‐II; Lck‐Cre × *Perp* ^fl/fl^ mice were injected into lethally irradiated recipient mice via tail veins, respectively. Chimeric mice were euthanized and analyzed 6 weeks after reconstitution.

### 2.3 Reagents and Antibodies

The antibodies were from BioLegend (TCRβ‐PE [H57‐597], CD5‐PE‐Cy7 [53‐7.3], CD8‐BV650 [53‐6.7], CD44‐APC [IM7], CD25‐BV421 [PC61], CD69‐PE‐Cy7 [H1.2F3], c‐Kit‐PE‐Cy7 [2B8], CD27‐BV421 [LG.3A10], Helios‐PE‐Cy7 [22F6], CD24‐BV421 [M1/69], and Qa‐2‐APC [695H1‐9‐9]); and from BD Pharmingen (CD4‐FITC [RM4‐5], Foxp3‐PE [R16‐715], CCR7‐AF647 [4B12], Vβ8.1/8.2‐PE [MR5‐2], Vβ2‐PE [B20.6], Vβ12‐PE [MR11‐1], IFN‐γ‐PE [XMG1.2], IL‐17A‐AF647 [TC11‐18H10], Biotin Mouse Lineage Panel [TER‐119, RB6‐8C5, RA3‐6B2, M1/70, 145‐2C11], purified anti‐mouse CD28 [37.51], and purified anti‐mouse CD3e [145–2C11]). The antibody to cleaved caspase‐3 (5A1E) was from Cell Signaling. The mouse 1G12 antibody, which is specific for TCR^3A9^, was isolated from an anti‐clonotype culture supernatant (DSHB Hybridoma Product 1G12), followed by APC conjugation with a rat anti‐mouse IgG1.

The Cytofix/Cytoperm Fixation and Permeabilization Solution, Transcription Factor Buffer Set, and PE Annexin V Apoptosis Detection kit were from BD Pharmingen. The CellTrace CFSE Cell Proliferation kit and Cell Stimulation Cocktail (plus protein transport inhibitors) were from Thermo Fisher Scientific.

### 2.4 Flow Cytometry

For direct staining, thymocytes or cultured cells were washed with cold PBS containing 1% w/v bovine serum albumin (BSA, BioFroxx), and then treated with a murine Fc Blocking Reagent (BD Pharmingen). Then, the thymocytes were incubated with fluorescence‐conjugated antibodies in PBS/1% BSA for 30 min at 4°C. For intracellular staining, fixation and permeabilization were performed using the Fixation/Permeabilization Solution kit with Cell Stimulation Cocktail (plus protein transport inhibitors) or the Transcription Factor Buffer Set (BD Pharmingen), followed by washing twice with cold PBS. Data were acquired with a Novocyte Quanteon Flow Cytometer (Agilent) and were analyzed using NovoExpress software (Agilent).

### 2.5 OT‐II Negative Selection Assay

The OT‐II negative selection assay was performed as described by Kim et al. (2021). In this assay, OT‐II; Lck‐Cre × *Perp* ^fl/+^ and OT‐II Tg; Lck‐Cre × *Perp* ^fl/fl^ mice were given intraperitoneal injections of 1 mg of the OVA_323–339_ peptide or the control peptide (OVA_257–264_) in 500 μL of PBS. Both peptides were from ChinaPeptides. The thymocytes were harvested after 72 h for analysis by flow cytometry (see above).

### 2.6 Immunoblotting Assay

Thymic CD4SP T‐cells were enriched from thymocytes of OT‐II; Lck‐Cre × *Perp* ^fl/+^ and OT‐II; Lck‐Cre × *Perp* ^fl/fl^ mice with OVA_323–339_ for 72 h. The purified CD4^+^ T cells from spleens were sorted on a FACSJazz sorter (BD Biosciences). Expression of BIM, cleaved caspase‐3, cleaved caspase‐9, PAG608, cytosolic cytochrome c, SMAC, BCL2, Bcl‐xL, SCOTIN, SIAH1 and PUMA proteins in each subset was detected with anti‐BIM antibody (#2933, Cell Signaling), anti‐cleaved caspase‐3 antibody (#9661, Cell Signaling), anti‐cleaved caspase‐9 antibody (#9509, Cell Signaling), anti‐PAG608 antibody (#10504–1‐AP, Proteintech), anti‐cytochrome c antibody (#4272, Cell Signaling), anti‐SMAC antibody (#15108, Cell Signaling), anti‐BCL2 antibody (#3498, Cell Signaling), anti‐Bcl‐xL antibody (#2762, Cell Signaling), anti‐SCOTIN antibody (#sc‐390,725, Santa Cruz), anti‐SIAH1 antibody (#PA5‐88583, Thermo Fisher), anti‐PUMA antibody (#98672, Cell Signaling), anti‐P16 antibody (#23200, Cell Signaling), and anti‐21 antibody (#37543, Cell Signaling). As the secondary antibody, anti‐rabbit IgG (#7074, Cell Signaling) or anti‐mouse IgG (#58802, Cell Signaling) was used. Signals were visualized with SuperSignal West Pico PLUS Chemiluminescent Substrate (Thermo Fisher) and analyzed by the ChemiDoc Imaging System (Bio‐Rad).

### 2.7 TCR Sequencing

RNA samples were analyzed by high‐throughput sequencing of TRB using the ImmuHub TCR profiling system at a deep level (ImmuQuad Biotech, Hangzhou China). Briefly, a 5′ RACE unbiased amplification protocol was used. This protocol uses unique molecular barcodes (UMBs) introduced in the course of cDNA synthesis to control bottlenecks and to eliminate PCR and sequencing errors. Sequencing was performed on an Illumina NovaSeq system with PE150 mode (Illumina). One common adaptor with UMB was added on the 5′ of cDNA during the first‐strand cDNA synthesis, and one reverse primer corresponding to the constant (C) regions of each of the TRB was designed to facilitate PCR amplification of cDNA sequences in a less biased manner. The UMB attached to each raw sequence read was applied to correct PCR and sequencing errors correction and PCR duplicates removing. Map V, D, J, and C segments with NCBI and then extract CDR3 regions and assemble clonotypes for all clones. The resulting nucleotide and amino acid sequences of CDR3 of TRB were determined, and those with out‐of‐frame and stop codon sequences were removed from the identified TRB repertoire. We further defined amounts of each TRB clonotype by adding numbers of TRB clones sharing the same nucleotide sequence of CDR3.

### 2.8 Saliva Flow Rate Test

After the animals were anesthetized with an intraperitoneal injection of ketamine/diazepam (100 mg/kg and 5 mg/kg, respectively), saliva flow rates of the 14‐month‐old Lck‐Cre × *Perp* ^fl/fl^ mice were determined for a period of 10 min, as described previously (Xu et al. 2012).

### 2.9 Quantitative PCR

Total RNA was extracted from the synovium, spinal cord, salivary glands, kidney, and from sorted CD4^+^ T cells derived from synovium and spinal cord of 14‐month‐old Lck‐Cre × *Perp* ^fl/fl^ mice using TRIzol reagent Takara (*Takara* Biochemicals, Dalian, China), following the manufacturer's instructions. Subsequently, 2 μg total RNA of each sample was reversely transcribed to cDNA using the iScript Advanced cDNA Synthesis Kit (Bio‐Rad Laboratories), according to the manufacturer's protocol. The relative levels of T‐bet, ROR‐γt, and Foxp3 mRNA transcripts to GAPDH were determined by a Bio‐Rad CFX 96 Real‐time Detection System using iQ SYBR Green Supermix (Bio‐Rad Laboratories) and specific primers. The sequences of primers were forward 5′‐AGCAAGGACGGCGAATGTT‐3′ and reverse 5′‐GGGTGGACATATAAGCGGTTC‐3′ for T‐bet; forward 5′‐GACCCACACCTCACAAATTGA‐3′ and reverse 5′‐AGTAGGCCACATTACACTGCT‐3′ for ROR‐γt; forward 5′‐CCCATCCCCAGGAGTCTTG‐3′ and reverse 5′‐ACCATGACTAGGGGCACTGTA‐3′ for Foxp3; forward 5′‐CCCAACGCCCCGAACT‐3′ and reverse 5′‐GCAGAAGAGCTGCTACGTGAA‐3′ for P16; forward 5′‐GAACATCTCAGGGCCGAAAA‐3′ and reverse 5′‐CGTGGGCACTTCAGGGTTT‐3′ for P21; and forward 5′‐AGGTCGGTGTGAACGGATTTG‐3′ and reverse 5′‐TGTAGACCATGTAGTTGAGGTCA‐3′ for GAPDH. The levels of mRNA transcripts were normalized to GAPDH and analyzed by the 2^−ΔΔCt^ method.

### 2.10 Statistical Analysis

Quantitative data were presented as means ± standard deviations (SDs). GraphPad Prism version 6.0 was used for all statistical analyses. The nonparametric, unpaired Mann–Whitney *U*‐test was used to determine the significance of differences in the medians of the two groups. The significance of differences among three or more groups was assessed using the repeated measures ANOVA with the post hoc Bonferroni correction. A *p* value below 0.05 was considered significant. Quantitative data are presented as means ± SD, and P values from comparisons are indicated as *p < 0.05, **p < 0.01, or n.s., not significant.

## 3 Results

### 3.1 *Perp* Knock‐Out Disrupts Thymic T‐Cell Development

Mice with the p53 null phenotype undergo normal T‐cell development, but eventually develop thymic double‐positive T‐cell lymphoma (Dudgeon et al. 2014). However, blockade of αβ T cell development in some mutant strains (e.g., *Wip1*‐null and *Rpl22‐null*) can rescue these mice due to the upregulation of p53 (Anderson et al. 2007). The reason for this response is that the p53 signaling network has multiple and diverse inputs and downstream outputs (Hernandez Borrero and El‐Deiry 2021). The p53 protein binds to the promoter of the *Perp* gene and induces its transcription during apoptosis, but the *Perp* gene is silent during cell cycle arrest, senescence, and noncanonical functions (Roberts and Paraoan 2020). Recent studies showed that loss of *Perp* function disrupted peripheral T‐cell homeostasis and inhibited the caspase‐dependent intrinsic pathway of apoptosis (Zhou et al. 2018, 2020). However, the role of *Perp* in thymocyte development is mostly unknown.

**FIGURE 1:**
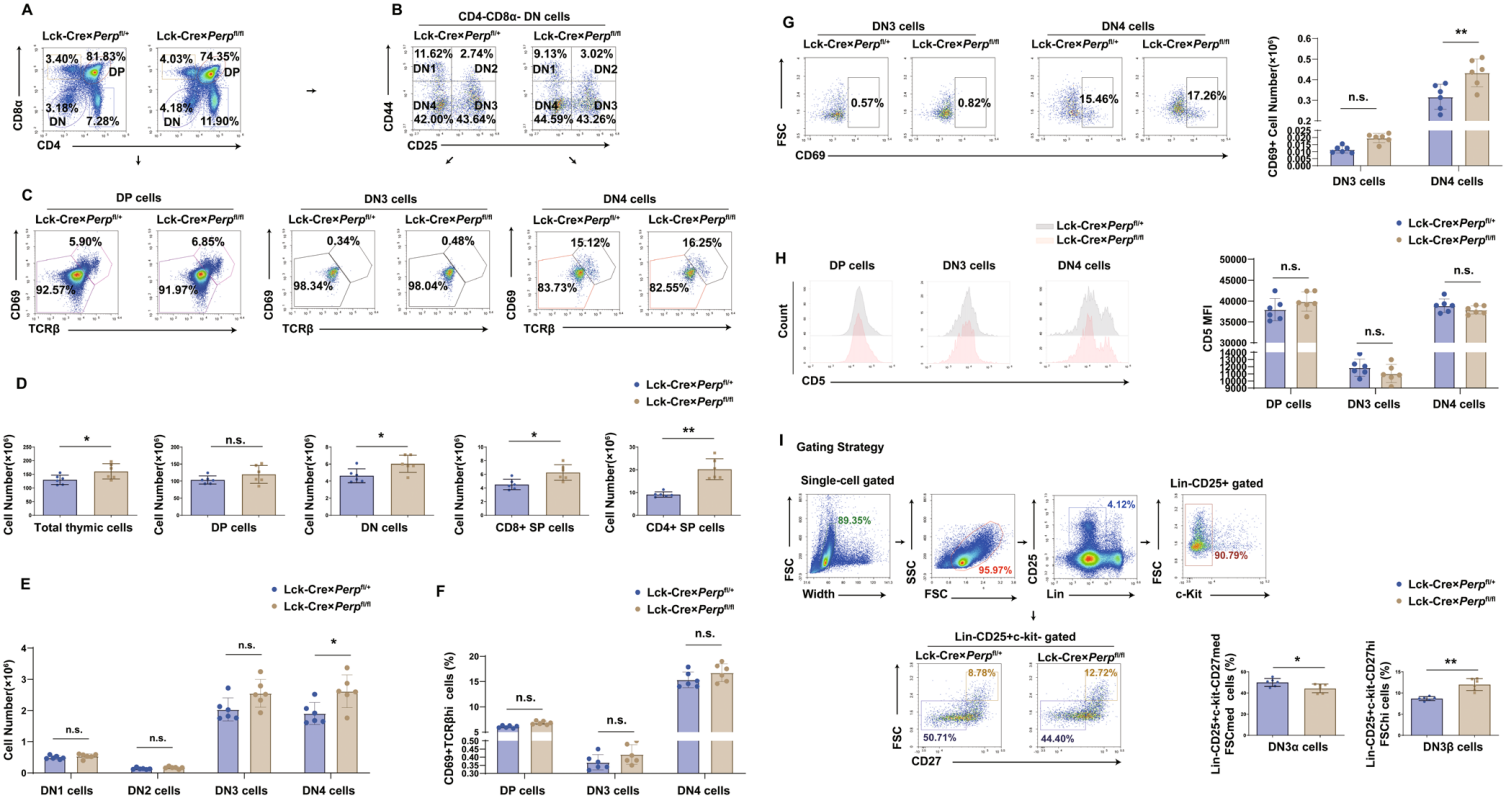
Lck‐Cre × *Perp* ^fl/fl^ mice have an increased percentage of CD4SP thymocytes. (A) Flow cytometry analysis of thymocytes in 6–8‐week‐old Lck‐Cre × *Perp* ^fl/+^ mice and Lck‐Cre × *Perp* ^fl/fl^ mice in the C57BL/6 background, with gates defining the CD4^−^CD8α^−^ (DN), CD4^+^CD8α^+^ (DP), CD4^+^CD8α^−^ (CD4SP), CD4^−^CD8α^+^ (CD8SP), and total thymocytes in both genotypes. (B) Percentages of CD44^+^CD25^−^ (DN1), CD44^+^CD25^+^ (DP2), CD44^−^CD25^+^ (DN3), and CD44^−^CD25^−^ (DN4) thymocytes in mice, analyzed as in A. (C) Percentages of CD69^+^TCRβ^hi^ DP, CD69^+^TCRβ^hi^ DN3, and CD69^+^TCRβ^hi^ DN4 thymocytes in mice, analyzed as in A. (D) Quantification of DP, DN, CD4SP, CD8SP, and total thymocytes in mice, analyzed as in A (6 mice per group). (E) Quantification of DN1, DN2, DN3, and DN4 in mice, analyzed as in A (6 mice per group). (F) Quantification of CD69^+^TCRβ^hi^ DP, CD4SP, and CD8SP thymocytes in mice, analyzed as in A (6 mice per group). (G) Flow cytometry analysis (left) and quantification (right) of CD69^+^ DN3 and DN4 thymocytes (6 mice per group; FSC, forward scatter). (H) Flow cytometry analysis of CD5 expression in different thymocyte populations. An overlay of Lck‐Cre × *Perp* ^fl/+^ mice (gray) and Lck‐Cre × *Perp* ^fl/fl^ mice (pink) is on the left, and quantification of CD5 expression is on the right (6 mice per group; MFI, mean fluorescence intensity). (I) Flow cytometry analysis (left) and quantification (right) of Lin‐CD25^+^c‐kit^−^CD27^hi^FSC^hi^ DN3α and Lin‐CD25^+^c‐kit^−^CD27^med^FSC^med^ DN3β thymocytes (6 mice per group). *p < 0.05, **p < 0.01, compared with littermate controls.

We therefore used floxing to develop conditional knock‐out mice for further investigation of this phenomenon. The results showed that *Perp* regulated thymic cellularity to a certain extent. In addition, *Perp* knock‐out induced a small increase in the total number of thymocytes. Cell counting demonstrated a nearly twofold increase in the number of CD4 single‐positive (SP) T‐cells in floxed (Lck‐Cre × *Perp* ^fl/fl^) mice compared with littermate control mice (Figure 1A,D). The percentage of DP cells was slightly decreased in the floxed mice; however, this difference was not statistically significant based on total cell numbers. In addition, the percentage of double negative (DN) thymocytes was also more than in the floxed mice (4.18% vs. 3.18%) (Figure 1D). Further analysis of CD44 and CD25 expression in DN cells demonstrated that the numbers of CD44^−^CD25^−^ DN4 thymocytes were a little higher in floxed mice than in littermate control mice (2.51 × 10^6^ vs. 1.92 × 10^6^; Figure 1B,E).

Signaling from pre‐TCRs is essential for the generation and subsequent expansion of thymocytes in the DN4 subset. We therefore analyzed cell surface expression of CD69/CD5 and intracellular expression of TCRβ in DN3, DN4, and DP thymocytes to assess the level of pre‐TCR signaling. The presence of this signaling in floxed mice indicated that *Perp* knock‐out did not alter the percentage of CD69^hi^ TCRβ^hi^ cells among DP, DN3, and DN4 thymocytes was unchanged (Figure 1C,F). However, there was an increased percentage of CD69+ DN4 thymocytes in the floxed mice, and CD69 expression was undetectable in the DN3 subset (Figure 1G). Analyses of CD69^+^ expression confirmed previous results that DN3 cells express little or no CD69 (Falk and Eichmann 2002), although CD69 was readily detected. Measurements of mean fluorescence intensity (MFI) demonstrated that *Perp* knock‐out did not affect the levels of CD5 in the DP, DN3, and DN4 thymocytes (Figure 1H). Furthermore, there was an increase in the percentage of postselection Lin‐CD25^+^c‐kit^−^CD27^hi^FSC^hi^ DN3β thymocytes in the floxed mice (Figure 1I). Taken together, these data show that *Perp* knock‐out and *p53* knock‐out impaired the development of thymic lymphocytes by different mechanisms.

### 3.2 *Perp* Differentially Regulates Survival of Thymic T‐Cell Subsets by Clonal Deletion

**FIGURE 2:**
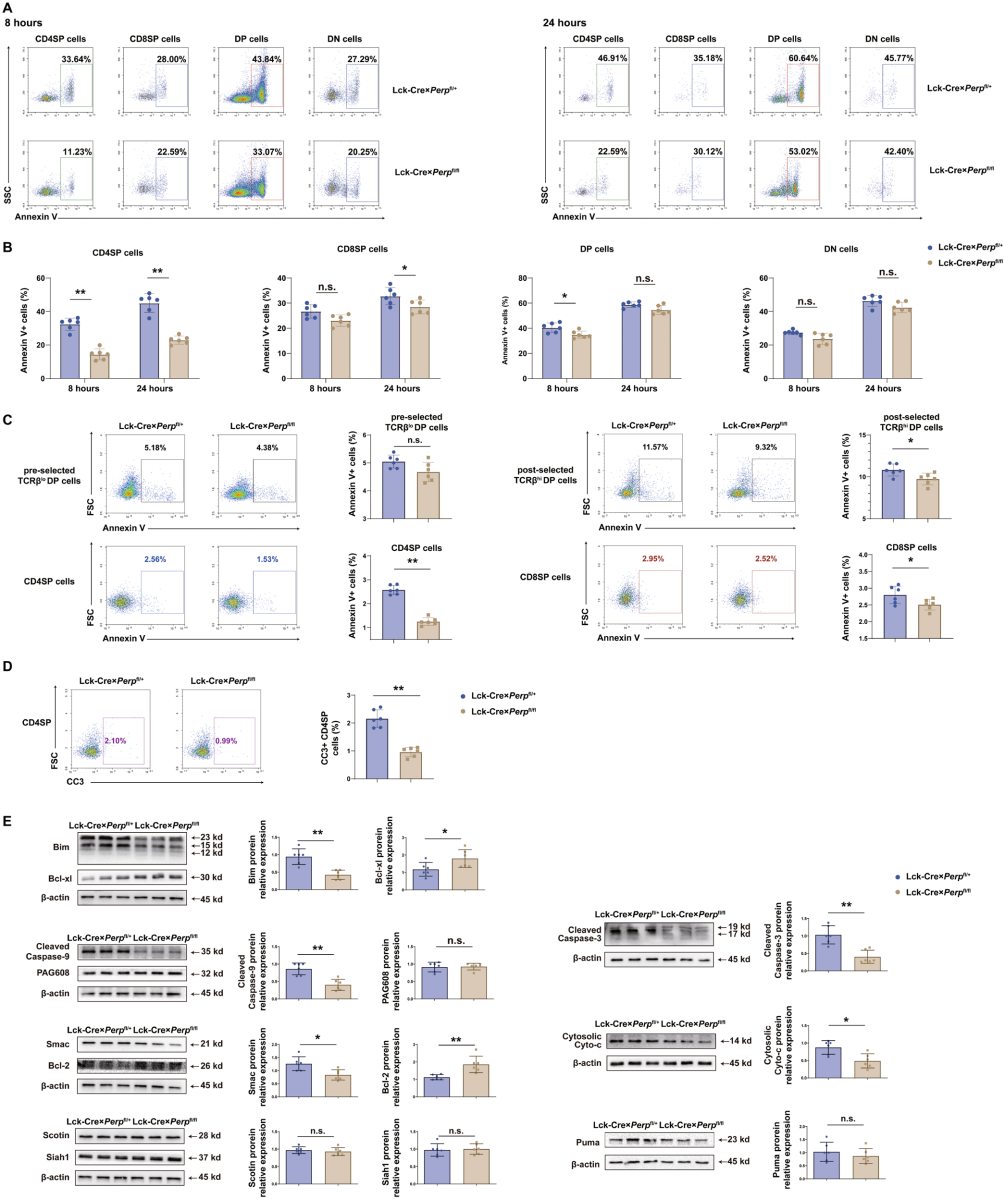
Deletion of *Perp* alters the survival of certain thymic T‐cell subsets during clonal deletion in vivo. (A) Flow cytometry analysis of annexin V in CD4SP, CD8SP, DP, and DN thymocytes after another 8 or 24 h of culture upon stimulation with double anti‐CD3e/CD28 (2 μg/mL each), and (B) quantification of these results (bottom, 6 mice per group). (C) Annexin V staining of freshly isolated preselected TCRβ^lo^ DP, postselected TCRβ^hi^ DP, CD4SP, and CD8SP thymocytes from 6 to 8‐week‐old Lck‐Cre × *Perp* ^fl/+^ and Lck‐Cre × *Perp* ^fl/fl^ mice, quantification of these results (left, 6 mice per group). (D) Flow cytometric detection of cleaved caspase‐3 (CC3) in CD4SP thymocytes and statistical quantification of CC3^+^ CD4SP thymocytes frequencies. (E) Western blotting (left rows) of apoptosis‐related markers in thymic CD4SP T‐cell lysates from OT‐II; Lck‐Cre × *Perp* ^fl/+^ and OT‐II; Lck‐Cre × *Perp* ^fl/fl^ mice after OVA_323–339_ injection, and densitometric analysis (right rows) of these results (6 mice per group, β‐actin: Loading control). *p < 0.05, **p < 0.01, compared with littermate controls.

T‐cell development in the thymus includes the processes of proliferation, maturation, and apoptosis, and extensive proliferation is responsible for maintaining the magnitude of distinct thymic T‐cell subpopulations (Hamm et al. 2025). However, apoptosis eliminates more than 95% of T‐cell precursors. Dysregulation of thymocyte apoptosis during clonal deletion leads to the escape of an excess of auto‐reactive T‐cells to the periphery, and the subsequent development of autoimmunity (Hojo et al. 2019). Thus, we examined the effect of TCR‐crosslinking on the induction of apoptosis of thymocytes from mice with different genotypes by performing ex vivo experiments. We first cultured thymocytes from littermate controls and floxed mice for 8 h or 24 h upon anti‐CD3/CD28 stimulation, and then evaluated thymocyte survival ex vivo. Flow cytometric analysis showed that *Perp* knock‐out decreased apoptosis to different degrees in different thymocyte subsets, with the largest effect on the CD4SP T‐cell subsets (Figure 2A,B). Moreover, we performed ex vivo apoptosis analysis on freshly isolated thymocytes using Annexin V staining; the percentage of Annexin V^+^ CD4SP T‐cell subsets in the floxed mice was significantly lower than that in littermate controls. There was also a decreased percentage of Annexin V^+^ postselected TCRβ^hi^ DP subsets in the floxed mice (Figure 2C). These results suggested that *Perp* may affect the survival of thymic T‐cell subsets by promoting clonal deletion, especially T cells in the CD4SP subset. Moreover, the frequency of cleaved caspase‐3 (CC3)‐positive cells within the CD4SP T‐cell subset of thymus was assessed by flow cytometry. The analysis revealed a significant decrease in the frequency of CC3^+^ CD4SP T‐cells in floxed mice compared to their littermate controls (Figure 2D). Our ex vivo and in vivo results suggested that CD4SP thymocytes in floxed mice were highly resistant to TCR crosslinking, an important step in the induction of apoptosis. Our previous research found that the *Perp*‐mediated regulation of Th17‐cell apoptosis was mediated by proteins in the BCL‐2 family (Zhou et al. 2018), a group of proteins that regulate many critical cell death checkpoints during immune development (Opferman 2008). We therefore investigated the effect of *Perp* knockout on BCL2 family members after TCR stimulation. More specifically, we isolated thymic CD4SP T‐cells from OT‐II; Lck‐Cre × *Perp* ^fl/+^ and OT‐II; Lck‐Cre × *Perp* ^fl/fl^ mice with OVA_323–339_ for 72 h, and then used western blotting to measure the expression of SMAC, BIM, BCL‐XL, BCL2, cleaved caspase‐3, PUMA, cleaved caspase‐9, and cytosolic cytochrome c. The results showed there were decreased levels of BIM, cleaved caspase‐3, cleaved caspase‐9, cytosolic cytochrome c, and SMAC in OT‐II Tg *Perp* ^−/−^ CD4SP thymocytes treated with OVA_323–339_ compared with OT‐II Tg *Perp* ^+/+^ CD4SP thymocytes. The loss of *Perp* also led to an obvious increase in BCL2 and BCL‐XL expression, the proteins associated with TCR crosslinking. However, *Perp* ^−/−^ did not affect the levels of PUMA in CD4SP thymocytes after stimulation (Figure 2E). We also used western blotting to examine the expression of all downstream proapoptotic genes of the p53 family in CD4SP thymocytes upon TCR crosslinking to determine whether other downstream proapoptotic genes may compensate for the lack of *Perp*. The results showed that *Perp* knock‐out had no effect on the expression of PAG608, SCOTIN, and SIAH1 in CD4SP thymocytes (Figure 2E).

### 3.3 *Perp* Knock‐Out Rescues OT‐II TCR+ Thymocytes From Negative Selection and Depends on TCR Affinity

**FIGURE 3:**
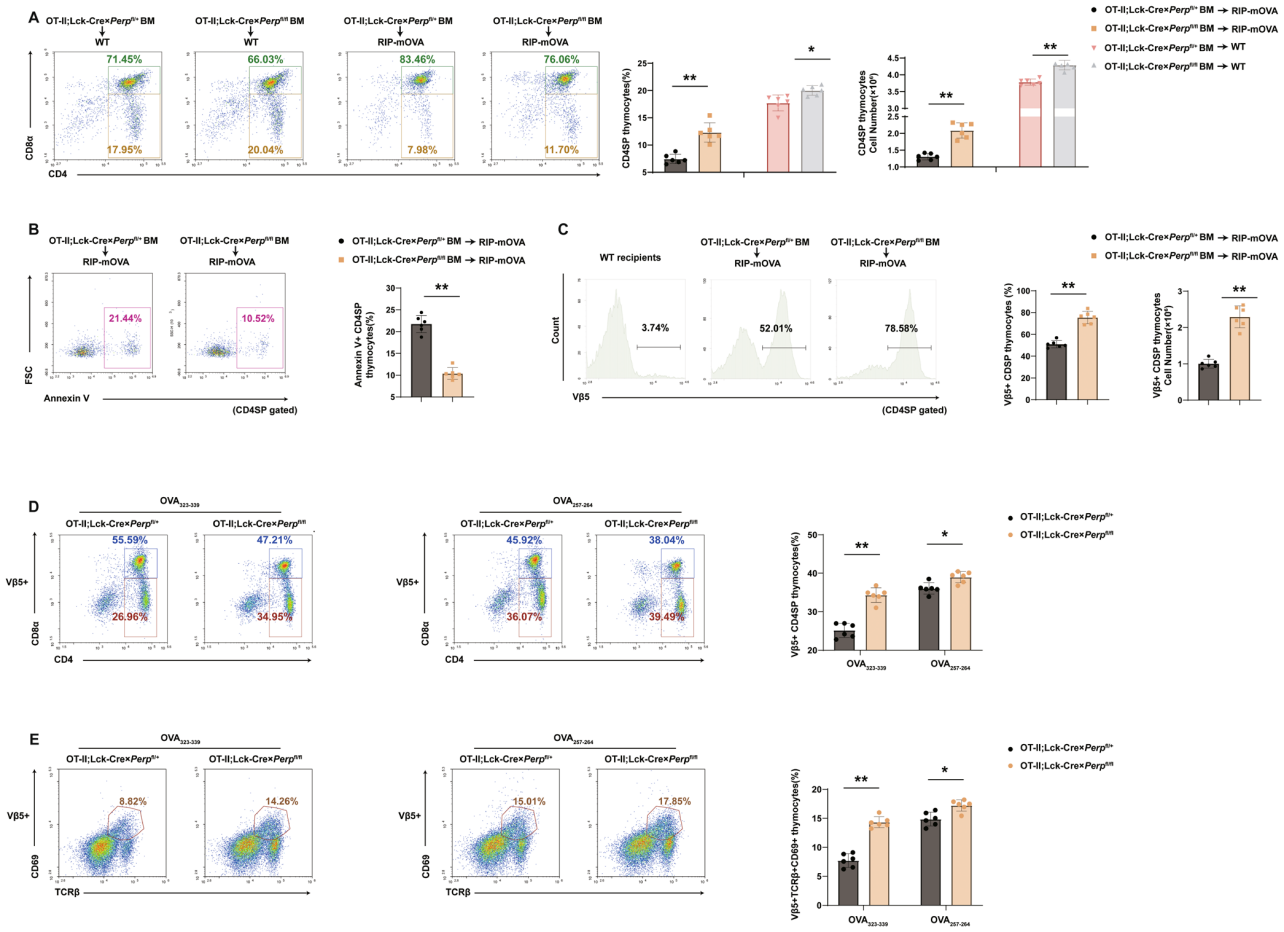
*Perp* is essential for depleting high‐affinity TCR clones in OT‐II × Rip‐mOVA Tg system. Schematic of transplantation of *Perp* ^+/+^ or *Perp* ^−/−^ OT‐II bone marrow into lethally irradiated wild type or RIP‐mOVA recipients. (A) Percentages of CD4SP thymocytes in mice were analyzed. (B) Percentages and numbers of Annexin V^+^ CD4SP thymocytes in mice, analyzed as in A. (C) Percentages and numbers of Vβ5^+^ CD4SP thymocytes in mice, analyzed as in A. (D) Flow cytometry analysis of thymocytes in 6–8‐week‐old OTII TCR transgenic mice and OTII; Lck‐Cre × *Perp* ^fl/fl^ mice according to the expression of CD4, CD8α, Vβ5, TCRβ, and CD69 after intraperitoneal administration of OVA_323–339_ or OVA_257–264_ (control) for 72 h. (E) Quantification of Vβ5^+^ CD4SP and Vβ5^+^TCRβ^+^CD69^+^ DP thymocytes in mice, analyzed as in D (6 mice per group). *p < 0.05, **p < 0.01, compared with littermate controls.

The presence of abundant CD4SP thymocytes in Lck‐Cre × *Perp* ^fl/fl^ mice led us to hypothesize that *Perp* may contribute to clonal deletion during thymic negative selection. We therefore examined whether *Perp* is required for the deletion of high‐affinity TCR clones in vivo by use of the OT‐II × RIP‐mOVA Tg system. We generated chimeric mice by transferring bone marrow cells of OT‐II; Lck‐Cre × *Perp* ^fl/+^ mice or OT‐II; Lck‐Cre × *Perp* ^fl/fl^ mice into irradiated RIP‐mOVA Tg mice. In OT‐II × Rip‐mOVA Tg system, we observed efficient negative selection of WT OT‐II T cells, as evidenced by a significant decrease in Vβ5 positive CD4SP T‐cell subsets. However, *Perp* deficiency rescued the survival of OT‐II CD4SP thymocytes of RIP‐mOVA mice and demonstrated significantly higher frequencies and numbers than *Perp* ^+/+^ OT‐II Tg CD4SP thymocytes (Figure 3A). Annexin V staining also revealed a decrease in apoptosis of *Perp*‐deficient CD4SP T‐cell subsets (Figure 3B). Further analysis of Vβ5 expression on CD4SP thymocytes revealed a greater than two‐fold increase in the number of TCR Vβ5^+^ cells within this subset (Figure 3C).

Moreover, the CD4SP thymocytes in these OT‐II Tg mice express Vα2/Vβ5 TCR, and this receptor primarily recognizes chicken ovalbumin‐derived peptide (OVA_323–339_) that is presented by I‐A^b^ MHC II (Hojo et al. 2019). Thus, we generated OT‐II; Lck‐Cre × *Perp* ^fl/fl^ mice by crossing, administered intraperitoneal injections of OVA_323–339_ or the control peptide (OVA_257–264_), and used OT‐II; Lck‐Cre × *Perp* ^fl/+^ mice as an additional control. Injection of OVA_323–339_ led to a significant depletion of CD4SP cells among control thymocytes in the OT‐II Tg mice (26.96% vs. 36.07%), and *Perp* knock‐out blocked this effect (Figure 3D). Staining of thymocytes with anti‐TCRβ and anti‐CD69 antibodies demonstrated that *Perp* significantly reversed the deletion of postselection thymocytes caused by OVA_323–339_ (Figure 3E). However, thymic positive selection was normal in Lck‐Cre × *Perp* ^fl/fl^ mice (Figure S1). Taken together with our results that examined the apoptosis and the TCR Tg model, these results indicate that *Perp* plays an important role in the deletion of high‐affinity TCR clones during thymic negative selection ex vivo and in vivo.

### 3.4 Lck‐Cre × *Perp* ^fl/fl^ Mice Have Expanded Helios^+^ CD4SP Thymocyte Populations

**FIGURE 4:**
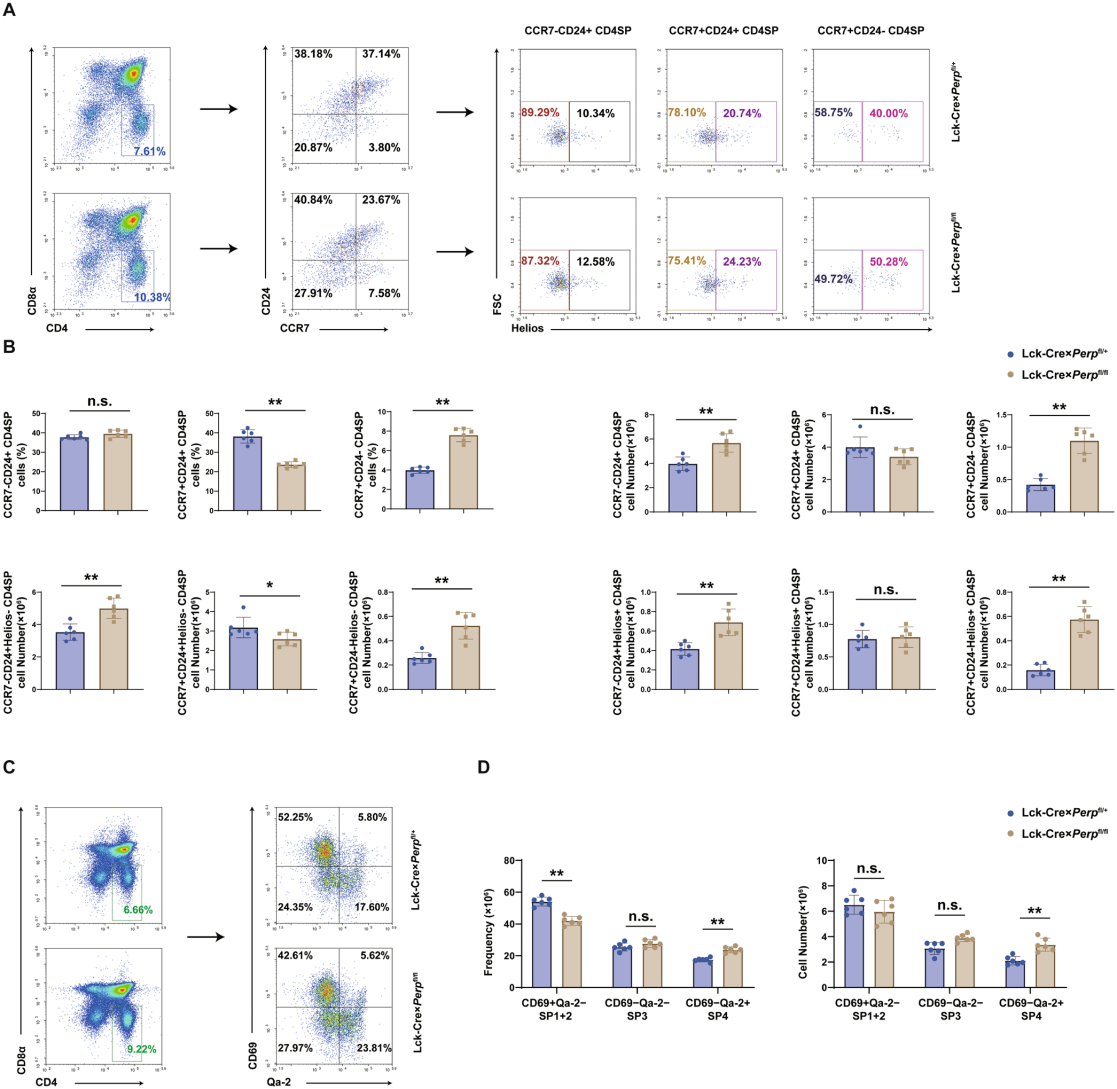
Lck‐Cre × *Perp* ^fl/fl^ mice accumulate Qa‐2^+^ CD4SP thymocytes. (A) Flow cytometry analysis of thymocytes in 6–8‐week‐old Lck‐Cre × *Perp* ^fl/+^ mice and Lck‐Cre × *Perp* ^fl/fl^ mice according to the expression of CD4, CD8α, CCR7, CD24, and Helios. (B) Quantification of CCR7^−^CD24^+^ CD4SP, CCR7^+^CD24^+^ CD4SP, and CCR7^+^CD24^−^ CD4SP thymocytes in mice, analyzed as in A (6 mice per group). (C) Flow cytometry analysis of thymocytes in 6–8‐week‐old Lck‐Cre × *Perp* ^fl/+^ and Lck‐Cre × *Perp* ^fl/fl^ mice according to the expression of CD4 and CD8α, Qa‐2, and CD69. (D) Quantification of CD69^+^Qa‐2^−^ (SP1 + SP2), CD69^−^Qa‐2^−^ (SP3), and CD69^−^Qa‐2^+^ (SP4) thymocytes, analyzed as in C (6 mice per group). *p < 0.05, **p < 0.01, compared with littermate controls.

SP thymocytes are normally subjected to negative selection in the medulla, and this eliminates autoreactive T‐cells that bind strongly to the antigens on mTECs. However, apoptosis‐deficient SP thymocytes may escape this thymic clonal deletion, thereby skewing the population of mature thymocytes. To monitor the number and maturation of CD4SP thymocytes undergoing negative selection, we measured Helios expression in three subsets of CD4SP thymocytes: (*i*) the least mature CCR7^−^CD24^+^ cells, (*ii*) the semi‐mature CCR7^+^CD24^+^ cells, and (*iii*) the most mature CCR7^+^CD24^−^ cells (Daley et al. 2013). The flow cytometry results showed that the percentage and number of CCR7^+^CD24^−^ CD4SP thymocyte subsets were significantly greater in the floxed mice than in littermate control mice (Figure 4A,B). Remarkably, there was a nearly three‐fold increase in the number of Helios^+^CCR7^+^ CD24^−^ CD4SP T‐cells in the floxed mice compared with the littermate control mice (Figure 4B). This increase in the number of Helios‐marked CD4SP thymocytes suggests that escape from negative selection may account for the excess of autoreactive T‐cells. Additionally, we used other lineage markers (CD4, CD8α, CD69, and Qa‐2) for the CD4SP subsets, and then analyzed cells at each gating (Aili et al. 2018). Consistent with our previous results, the floxed mice had substantially increased in the CD69^−^Qa‐2^+^ (SP4) subsets of CD4SP thymocytes (Figure 4C,D). However, *Perp* knockout does not impair the generation or function of thymic regulatory T (Treg) cells (Figure S2). Furthermore, Lck‐Cre × *Perp* ^fl/fl^ mice have normal thymocyte proliferation and an increased percentage of CD4SP thymocytes (Figure S3).

### 3.5 Lck‐Cre × *Perp* ^fl/fl^ Mice Demonstrate a Skewed TCR Repertoire in Peripheral CD4^+^ T Cells, Indicative of a Predisposition for Autoimmunity

**FIGURE 5:**
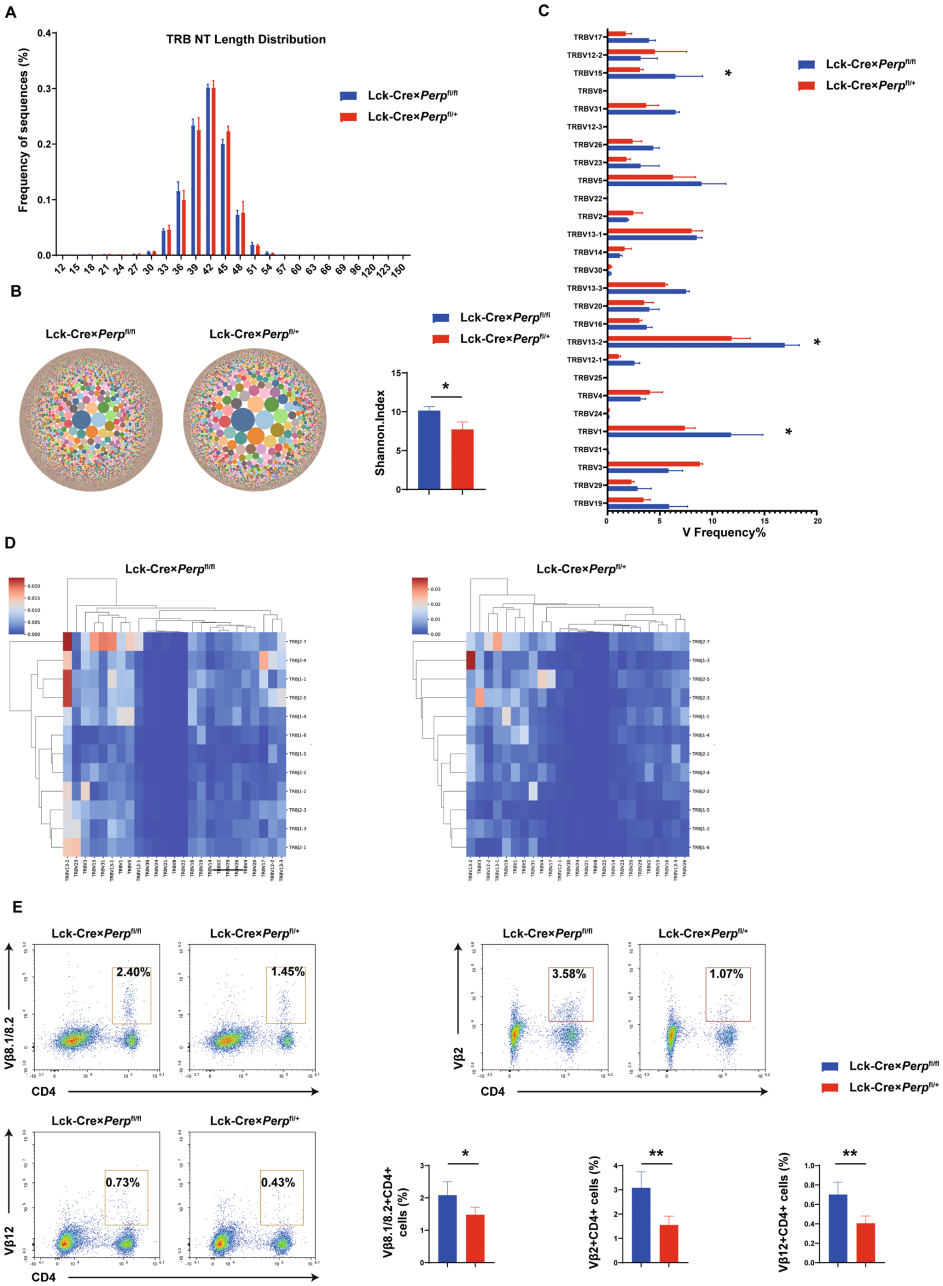
Lck‐Cre × *Perp* ^fl/fl^ mice exhibit a biased TCR repertoire in peripheral CD4^+^ T cells. (A) The profile of CDR3 length distribution. (B) The bubble diagram illustrates the frequency of each clone species; a greater number and density of bubbles correspond to a higher number of clone species in the sample. Shannon index between Lck‐Cre × *Perp* ^fl/+^ and Lck‐Cre × *Perp* ^fl/fl^ mice (*n* = 3). (C) The graph depicts the expression frequency of clones with significant differences between 12‐week‐old Lck‐Cre × *Perp* ^fl/+^ and Lck‐Cre × *Perp* ^fl/fl^ mice (*n* = 3). (D) Frequencies of V and J gene fragment combinations in the representative sample were determined and heat maps were constructed, with warm colors (red) representing high frequencies and cool colors (blue) representing low frequencies. The connection lines indicate clustering relationships, where higher similarity in V/J gene use frequency corresponds to closer clustering. (E) Representative flow cytometry pseudo‐color plots showing intracellular staining for Vβ8.1/Vβ8.2, Vβ2, and Vβ12 in CD4^+^ T cells isolated from spleens and statistical quantification of Vβ8.1/Vβ8.2^+^CD4^+^, Vβ2^+^CD4^+^, and Vβ12^+^CD4^+^ T‐cells frequencies. *p < 0.05, **p < 0.01, compared with littermate controls.

A consequence of defective negative selection in Lck‐Cre × *Perp* ^fl/fl^ mice might be manifested as a skewed TCR repertoire, resulting from a higher frequency of the most autoreactive T cell clones. To study changes in the TCR repertoire, we purified peripheral CD4+ T cells from 12‐week‐old Lck‐Cre × *Perp* ^fl/+^ and Lck‐Cre × *Perp* ^fl/fl^ mice, sequenced the TRBV‐encoding mRNAs, and performed unbiased PCR expansion. We first determined the TRBV CDR3 length distribution of peripheral CD4^+^ T cells isolated from Lck‐Cre × *Perp* ^fl/+^ and Lck‐Cre × *Perp* ^fl/fl^ mice. Spectratyping of 24 TRBV families revealed a normal distribution of CDR3 lengths between Lck‐Cre × *Perp* ^fl/+^ and Lck‐Cre × *Perp* ^fl/fl^ mice (Figure 5A). TCR diversity was slightly higher in Lck‐Cre × *Perp* ^fl/fl^ mice compared to Lck‐Cre × *Perp* ^fl/+^ mice, as determined by Shannon index analysis (9.646 ± 0.806 vs. 7.643 ± 0.838, *n* = 3). The size of the bubble represents the frequency of the clone within the population, and the number within the bubble represents the clone count (Figure 5B). To further investigate this, we compared the relative usage frequency of TRBV segments between Lck‐Cre × *Perp* ^fl/+^ and Lck‐Cre × *Perp* ^fl/fl^ mice. Some VJ combinations, such as TRBV13‐2, TRBV1, and TRBV15, appeared with high frequency in Lck‐Cre × *Perp* ^fl/fl^ (Figure 5C). Previous studies have reported that TRBV13‐2 (Vβ8.2) and TRBV1 (Vβ2) seemed to initiate the development of murine autoimmunity models (Song et al. 2022). Hierarchical clustering of the representative samples based on public TCR clones also revealed higher heterogeneity in Lck‐Cre × *Perp* ^fl/fl^ mice (Figure 5D). Furthermore, we performed additional flow cytometry experiments to detect the frequencies of TRBV13‐2 (Vβ8.2), TRBV1 (Vβ2), and TRBV15 (Vβ12) of splenic CD4^+^ T‐cells from Lck‐Cre × *Perp* ^fl/+^ and Lck‐Cre × *Perp* ^fl/fl^ mice. Flow cytometry data showed that the percentages of splenic Vβ8.1/Vβ8.2^+^CD4^+^, Vβ2^+^CD4^+^, and Vβ12^+^CD4^+^ T‐cells were significantly greater in the floxed mice than in littermate control mice (Figure 5E). Collectively, these findings validate and corroborate the TCR sequencing data.

### 3.6 Middle‐Aged Lck‐Cre × *Perp* ^fl/fl^ Mice Have T Cell‐Mediated Autoimmune Arthritis

**FIGURE 6:**
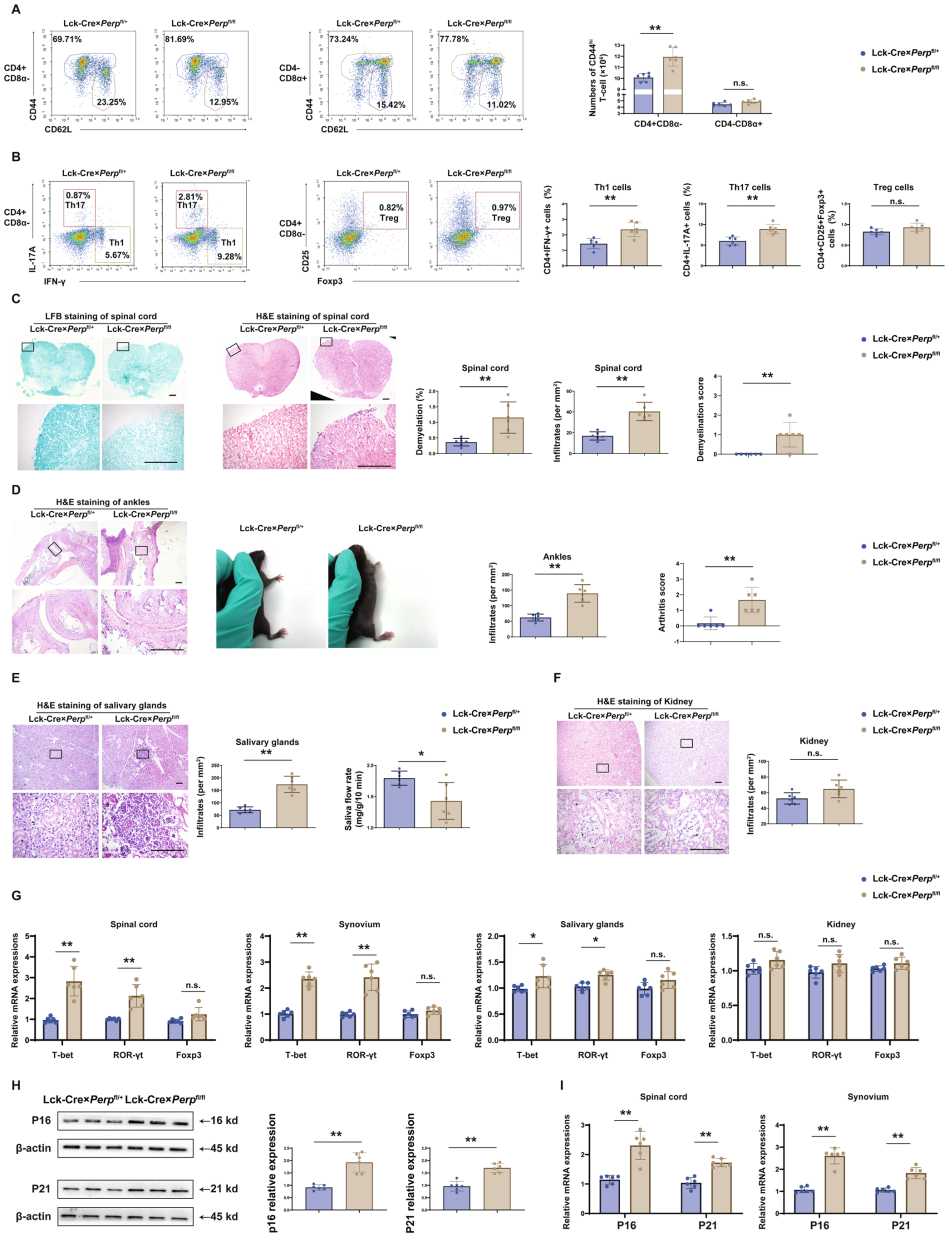
Middle‐aged Lck‐Cre × *Perp* ^fl/fl^ mice develop spontaneous autoimmunity. (A) Flow cytometry analysis of thymocytes in 14‐month‐old Lck‐Cre × *Perp* ^fl/+^ mice and Lck‐Cre × *Perp* ^fl/fl^ mice according to the expression of CD4, CD8α, CD44, and CD62L. Quantification of CD4^+^CD8α^−^CD44^hi^ activated T‐cells in mice, analyzed as in A (6 mice per group). (B) Representative flow cytometry pseudo‐color plots showing intracellular staining for IL‐17A, IFN‐γ, and Foxp3 in CD4^+^ T cells isolated from spleens and statistical quantification of Th17 (CD4^+^IL‐17A^+^) and Th1 (CD4^+^IFN‐γ^+^), and Treg (CD4^+^CD25^+^Foxp3^+^) frequencies. (C) Histochemistry of demyelination and inflammatory infiltrates in the spinal cords of different mice (staining by luxol blue or hematoxylin & eosin, transverse sections, 6 mice per group). Demyelination was assessed using the following criteria: 0, no paralysis; 1, flaccid tail; 2, impaired righting reflex and/or gait; 3, partial hind limb paralysis; 4, complete hind limb paralysis. (D) Histochemistry of inflammatory infiltrates in the ankles of different mice (staining by hematoxylin & eosin, longitudinal sections, 6 mice per group). The arthritis severity was evaluated in average combined score of all four paws of the mice by three independent observers. The severity was scored as follows: 0, normal; 1, erythema and mild swelling; 2, erythema and slight swelling extending from the ankle to metatarsal joints; 3, erythema and moderate swelling; 4, erythema and severe swelling encompass the ankle, foot and digits. (E) Histochemistry of inflammatory infiltrates in the salivary glands of different mice (staining by hematoxylin & eosin, cross sections, 6 mice per group). Saliva flow rates of mice in different groups (6 mice per group). (F) Histochemistry of inflammatory infiltrates in the kidneys in different mice (staining by hematoxylin & eosin, cross sections, 6 mice per group). (G) Quantitative real‐time PCR analysis of lineage‐specific transcription factors in synovium, spinal cord, salivary glands, and kidney, including T‐bet (Th1), ROR‐γt (Th17), and Foxp3 (Treg). Relative mRNA expression was normalized to GAPDH using the 2^−ΔΔCt^ method (*n* = 6 replicates per group). (H) Western blotting (left rows) of P16 and P21 in splenic CD4^+^ T cells lysates from middle‐aged Lck‐Cre × *Perp* ^fl/+^ and Lck‐Cre × *Perp* ^fl/fl^ mice, and densitometric analysis (right rows) of these results (6 mice per group, β‐actin: loading control). (I) Quantitative real‐time PCR analysis of P16 and P21 in sorted CD4^+^ T cells derived from synovium and spinal cord. Relative mRNA expression was normalized to GAPDH using the 2^−ΔΔCt^ method (*n* = 6 replicates per group). *p < 0.05, **p < 0.01, compared with littermate controls.

*Perp* knock‐out rescued OT‐II TCR+ thymocytes from negative selection, and this depended on TCR affinity. The expanded high‐affinity TCR clones in floxed mice could leak into the peripheral circulation and then contribute to autoimmunity. We therefore examined whether defective negative selection of *Perp* ^−/−^ CD4SP thymocytes induced autoimmunity in middle‐aged mice. The results showed there was substantial accumulation of activated CD4^+^CD44^hi^ T‐cells in the spleens of floxed mice that were 14‐months‐old (Figure 6A). Flow cytometry data also showed that the percentages of splenic Th1 and Th17 T‐cells were significantly greater in the floxed mice than in littermate control mice (Figure 6B). This autoimmunity manifested as significant infiltration of inflammatory cells and demyelination in the spinal cord (Figure 6C). Moreover, the knee joints of these middle‐aged floxed mice had inflammatory cell infiltrates and synovial hyperplasia, with mild erythema and swelling of the hind paws (Figure 6D), infiltration of lymphocytes into the salivary glands, and a slight decrease in the rate of saliva flow (Figure 6E). Moreover, the transcription factors of CD4^+^ helper cells were measured by quantitative real‐time PCR, and the results showed that the expression levels of T‐bet (Th1) and ROR‐γt (Th17) were significantly upregulated in the synovium, spinal cord, and salivary glands of floxed mice, compared to littermate control mice (Figure 6G). All of these responses are signs of an autoimmune disorder.

Furthermore, senescence undermines immune surveillance and disrupts self‐tolerance mechanisms in diseases such as rheumatoid arthritis, systemic lupus erythematosus, and multiple sclerosis (Hu et al. 2026; Yin et al. 2025). We performed additional western blot experiments to detect the P16 and P21 protein level of splenic CD4^+^ T‐cells from middle‐aged Lck‐Cre × *Perp* ^fl/+^ and Lck‐Cre × *Perp* ^fl/fl^ mice. Western blot data showed that the P16 and P21 expression levels of splenic CD4^+^ T‐cells significantly increased in the floxed mice compared to littermate control mice (Figure 6H). In addition, P16 and P21 of sorted CD4^+^ T cells from synovium and spinal cord were measured by quantitative real‐time PCR, and the results showed that the expression levels of P16 and P21 were significantly upregulated in synovium and spinal cord of floxed mice compared to littermate control mice (Figure 6I).

## 4 Discussion

Appropriate regulation of apoptosis is critical for the deletion of self‐reactive T‐cells during thymic negative selection, a process that allows the development of immunological self‐tolerance (Ashby and Hogquist 2023; Sogkas et al. 2021). Previous studies have identified numerous factors that affect cell fate during T‐cell development (Rathmell et al. 2002; Shanmuganad et al. 2022). Our ex vivo and in vivo experiments showed there was significant accumulation of thymic CD4SP T‐cells in 6–8‐week‐old floxed mice (Lck‐Cre × *Perp* ^fl/fl^), and that *Perp* regulated the survival of thymic T‐cell subsets during clonal deletion, especially CD4SP T‐cells after TCR stimulation. Our experiments with Helios, a marker of strongly self‐reactive TCRs, demonstrated an expanded Helios^+^ CD4SP thymocyte population in floxed mice. We also found that *Perp* knock‐out rescued OT‐II TCR^+^ thymocytes from negative selection, and that this was dependent on TCR affinity. Consistent with previous observations, we found an excessive accumulation of activated CD4^+^ T‐cells (CD44^hi^CD62L^lo^) in the peripheral blood of floxed mice when they were 14‐months‐old, indicating a T cell‐mediated autoimmune arthritis (Zhou et al. 2020).

Our results demonstrated that *Perp* knock‐out disrupted the normal development of CD4^+^ and CD8^+^ T‐cell progenitors, with some variation among subtypes. Thymic CD4^+^ T‐cells from floxed mice had a substantial survival advantage compared with CD8^+^ T‐cells from the same mice during clonal deletion, and conditional knockout of *Perp* in mice significantly reduced apoptosis of thymic CD4SP T‐cells after 8 and 24 h of in vitro culture. As expected, there was a notable increase in the number and percentage of CD4SP T‐cells in floxed mice, even though *Perp* knock‐out had different effects on different thymic T‐cell subsets. Similarly, the absolute number of peripheral CD4^+^ T‐cells, but not CD8^+^ T cells, was also greater in floxed mice, as previously described (Zhou et al. 2020). There is evidence that the CD4/CD8 lineage of developing thymocytes affects the number of CD4SP and CD8SP T‐cells (Shinzawa et al. 2022). A strong and persistent MHC‐II‐restricted TCR signaling activates GATA3 (a transcription factor), which commits DP thymocytes toward the CD4 phenotype, and also induces the expression of a repressor (ThPOK) that prevents the expression of genes that are characteristic of CD8^+^ T‐cells (Zeidan et al. 2019). Conversely, a weak MHC‐I/TCR signal activates other transcription factors, such as RUNX3, that maintain the CD8^+^ T cell phenotype (Etzensperger et al. 2017; Karimi et al. 2021). We found no significant difference in the number and frequency of BrdU^+^ CD8SP and BrdU^+^ CD4SP cells in floxed mice at 1 day after BrdU injection. *Perp* knock‐out also did not affect the number of weakly self‐reactive Helios TCR^3A9+^ CD4SP T‐cells that were positively selected in 3A9 TCR mice. However, our results provide no evidence that *Perp* affected the CD4‐CD8 lineage in vivo. Previous research showed that stronger TCR signaling was critical for development of the CD4 lineage due to alterations in chromatin structure by certain genes (GATA3, Tox, c‐Myb, and ThPOK) that are associated with the CD4 helper T cell lineage (Shinzawa et al. 2022; Zeidan et al. 2019). Strong signaling from the interaction of the TCR with the MHC–peptide complex is therefore more likely to trigger apoptosis during thymic negative selection (Kappes et al. 2005; Singer et al. 2008). We observed that *Perp* knock‐out led to a partial but significant rescue of the high‐affinity TCR CD4^+^ T‐cell clone during thymic negative selection ex vivo and in vivo. We therefore propose that *Perp* ^−/−^ CD4SP T‐cells had the greatest resistance to apoptosis due to induction by high‐affinity TCR stimulation, and this may bias the thymic CD4^+^/CD8^+^ T‐cell ratio.

Our studies of Lck‐Cre × *Perp* ^fl/fl^ mice showed that thymic CD4SP cells increased significantly, but the number of CD4^+^CD8^−^Foxp3^+^ thymic Tregs only increased slightly and without statistical significance. Although TCR/CD28 costimulation is required for the generation of thymic Tregs, other studies demonstrated that γc family cytokines (IL‐2, IL‐4, IL‐7, and IL‐15) and TNF superfamily cytokines (GITRL, OX40L, and TNF‐α) were critical for the development of thymic Tregs due to their regulation of FOXP3 expression (Owen et al. 2019; Tang et al. 2022). Consequently, we speculate that the slight increase of thymic Tregs in the floxed mice was likely to be a compensatory response to defective negative selection and was independent of *Perp* knock‐out. In other words, a deficit in thymic negative selection does not inevitably lead to autoimmunity, because additional mechanisms can contribute to self‐tolerance (Cheru et al. 2023). It is important to note that aged Tregs are less efficient than young Tregs in suppressing effector T‐cell activation and proliferation (Mittelbrunn and Kroemer 2021). Considering previous studies together with our results, we suggest that the simultaneous presence of defective negative selection, excessive accumulation of activated CD4^+^ T‐cells, and aged Tregs in middle‐aged floxed mice were responsible for the development of autoimmunity.

*Perp* is a crucial tumor suppressor that is downregulated in a variety of cancers (Roberts and Paraoan 2020). Low expression of *Perp* leads to decreased apoptosis in cancer cells, as demonstrated by reductions in caspase activation and the expression of proapoptotic genes (Beaudry et al. 2010; Holmes et al. 2020). Similarly, our previous study demonstrated that *Perp* ^−/−^ Th17 cells were relatively resistant to activation‐induced cell death (AICD) in vitro, and that this was accompanied by inhibition of the caspase‐dependent intrinsic pathway of apoptosis (Zhou et al. 2018). We also found that peripheral *Perp* ^−/−^ CD4^+^ effector memory T‐cells undergoing lymphopenia‐induced proliferation had a competitive advantage over wild type cells due to their increased resistance to apoptosis in adoptive transfer assays (Zhou et al. 2020). The present study demonstrated that *Perp* knock‐out led to an obvious decrease in the apoptosis of CD4SP T‐cell subsets and a decreased level of activated caspase‐3. Conditional knockout of *Perp* in mice significantly reduced apoptosis of thymic CD4SP T‐cells ex vivo and in vivo. These results suggest that *Perp* may have an important role in regulating immune homeostasis and inflammatory diseases. These findings also explain why *Perp* knock‐out enhanced T‐cell infiltration and activation in the tumor microenvironment, and the induction of inflammation‐related genes that stimulate tumorigenesis.

Many studies have established that p53 is a key tumor suppressor that regulates the cell cycle, cell differentiation, cell proliferation, DNA repair, and energy metabolism. P53 also has antioxidant and antiangiogenic effects and functions in autophagy, aging, and apoptosis (Hassin and Oren 2023). The impact of p53 on the negative selection of thymic T cells is multifaceted and represents a shift from a direct cell‐death mediator to a master regulator of the thymic niche (Haines et al. 2006). While the terminal apoptosis of a self‐reactive thymocyte can proceed in the absence of p53, the medullary environment required to present the “self” and the genomic surveillance required to prevent aberrant clones are entirely dependent on p53 activity (Larsen and Bhandoola 2017). Unlike p53, which regulates multiple target genes that have diverse biological functions, the *Perp* gene, whose promoter is targeted by p53, only functions in the regulation of apoptosis‐related signaling. Our previous studies indicated that loss of *Perp* in T‐cells did not affect the differentiation of Th1, Th17, and Treg cells in vitro, and *Perp* knock‐out did not alter lymphopenia‐induced cell proliferation, including rapid spontaneous proliferation and slow homeostatic proliferation (Zhou et al. 2018, 2020). The results from these previous studies also suggest the presence of normal proliferation of CD4SP thymocytes in floxed mice. Our finding that *Perp* has a crucial role in cell survival suggests that targeting *Perp* or *Perp*‐related molecules may be effective for the treatment of autoimmune diseases.

In conclusion, our results demonstrated that mice with conditional *Perp*‐knock‐out had a defect in thymic negative selection and developed autoimmunity as they aged. However, whether *Perp* functions as a key checkpoint for T‐cell development and autoimmune diseases needs to be verified by restoring PERP protein production with recombinant adeno‐associated virus (rAAV)‐based gene therapy. The causes of *Perp* down‐regulation in the T‐cells of patients with various autoimmune diseases also need to be further examined. Finally, as a transmembrane protein, it is possible that *Perp* is a druggable target. These are all intriguing topics that deserve further exploration.

## Author Contributions

Yan Zhou: conceptualization; formal analysis; validation; investigation; funding acquisition; writing – original draft; writing – review and editing. Junrong Li: conceptualization; formal analysis; validation; investigation; writing – review and editing. Xiao Leng: formal analysis; validation; investigation. Jiao Shi: formal analysis; methodology. Gan Zhang: investigation; methodology. Shan Chen: investigation; methodology. Dong Liu: formal analysis; investigation; methodology. Qian Deng: formal analysis; investigation; methodology. Yan He: formal analysis; investigation; methodology. Guixiu Shi: conceptualization; resources. Ying Xu: conceptualization; data curation; formal analysis. Yuan Liu: conceptualization; resources; supervision; validation; visualization; methodology. Yantang Wang: conceptualization; resources; data curation; formal analysis; supervision; funding acquisition; validation; visualization; methodology; writing – original draft; project administration; writing – review and editing.

## Funding

This work was supported by National Natural Science Foundation of China, 81871300, 81901521. Natural Science Foundation of Sichuan Province, 2025 NSFSC2147. CMC Excellent‐talent Program, 2024kjTzn04. The Research Fund of Development and Regeneration Key Laboratory of Sichuan Province, 24FYYZ507.

## Conflicts of Interest

The authors declare no conflicts of interest.

## Supporting information


**Figure S1:** acel70514‐sup‐0001‐FiguresS1‐S3.docx. *Perp* knock‐out does not affect normal positive selection.
**Figure S2:** acel70514‐sup‐0001‐FiguresS1‐S3.docx. *Perp* deficiency does not alter the development of thymic Tregs.
**Figure S3:** Lck‐Cre × *Perp* ^fl/fl^ mice have normal thymocyte proliferation.

## Data Availability

The data that support the findings of this study are openly available in Mendeley Data at https://data.mendeley.com/preview/trh2w8pzc7?a=9dec6eaa‐2718‐472f‐a152‐e7e5f058ebe9, reference number, Doi: 10.17632/trh2w8pzc7.2.
