# Supplementary material for: Perp Deficiency Induces Defective Negative Selection and Autoimmune Arthritis in Aged Mice
Source: Aging Cell. 2026 Apr 23;25(5):e70514. doi: 10.1111/acel.70514 (PMC13104118; doi:10.1111/acel.70514)
Supplement: Supplementary file 1 — Figure S1: acel70514‐sup‐0001‐FiguresS1‐S3.docx. Perp knock‐out does not affect normal positive selection. Figure S2: acel70514‐sup‐0001‐FiguresS1‐S3.docx. Perp deficiency does not alter the development of thymic Tregs. Figure S3: Lck‐Cre × Perp fl/fl mice have normal thymocyte proliferation. [file ACEL-25-e70514-s001.docx]

Supplementary Materials

**Mice**

3A9 TCR Tg mice (strain No. 016132) were obtained from Jackson Laboratories (ME, USA). Lck-Cre×*Perp*^fl/fl^ mice were crossed with TCR Tg mice that expressed I-A^k^ MHC class II to generate 3A9;Lck-Cre×*Perp*^fl/fl^ mice.

**Treg suppressive function assay**

Splenocytes were isolated from 12-week-old Lck-Cre×*Perp*^fl/fl^ mice or Lck-Cre×*Perp*^fl/+^ mice. Then, erythrocyte-depleted splenocytes were prepared using an RBC lysis buffer, and stained with anti-CD4 and anti-CD25 antibodies. The resulting CD4^+^CD25^+^ Treg and CD4^+^CD25^−^ Teff cells were sorted on a FACSJazz sorter (BD Biosciences). The Teff cells were stained with carboxyfluoroscein succinimidyl ester (CFSE) using a standard protocol (Hawkins et al., 2007). Then 5 ×10^4^ Teff cells and 2.5×10^4^ Treg cells were co-cultured in a 96-well U-bottom plate with 5×10^4^ antigen presenting cells (APCs, bone marrow derived dendritic cells from C57BL/6 mice) that were inactivated by gamma irradiation, 1 µg/mL of anti-CD3ε, and 2 µg/mL of anti-CD28 in RPMI-1640 medium (total of 100 μL per well) for 72 h. CFSE fluorescence from the CD4^+^CD25^−^ Teff cells was measured using a Novocyte Quanteon flow cytometer.

**BrdU cell proliferation assay**

The BrdU incorporation assay was performed using the BrdU Cell Proliferation Assay kit (BioLegend). Briefly, Lck-Cre×*Perp*^fl/+^ mice and Lck-Cre×*Perp*^fl/fl^ mice received a single 1 mg injection of BrdU after 1, 2, 3, 4, 5, or 6 days. Duplicates of all thymocyte samples were analyzed on the same day.

**Supplementary figure and figure legends**


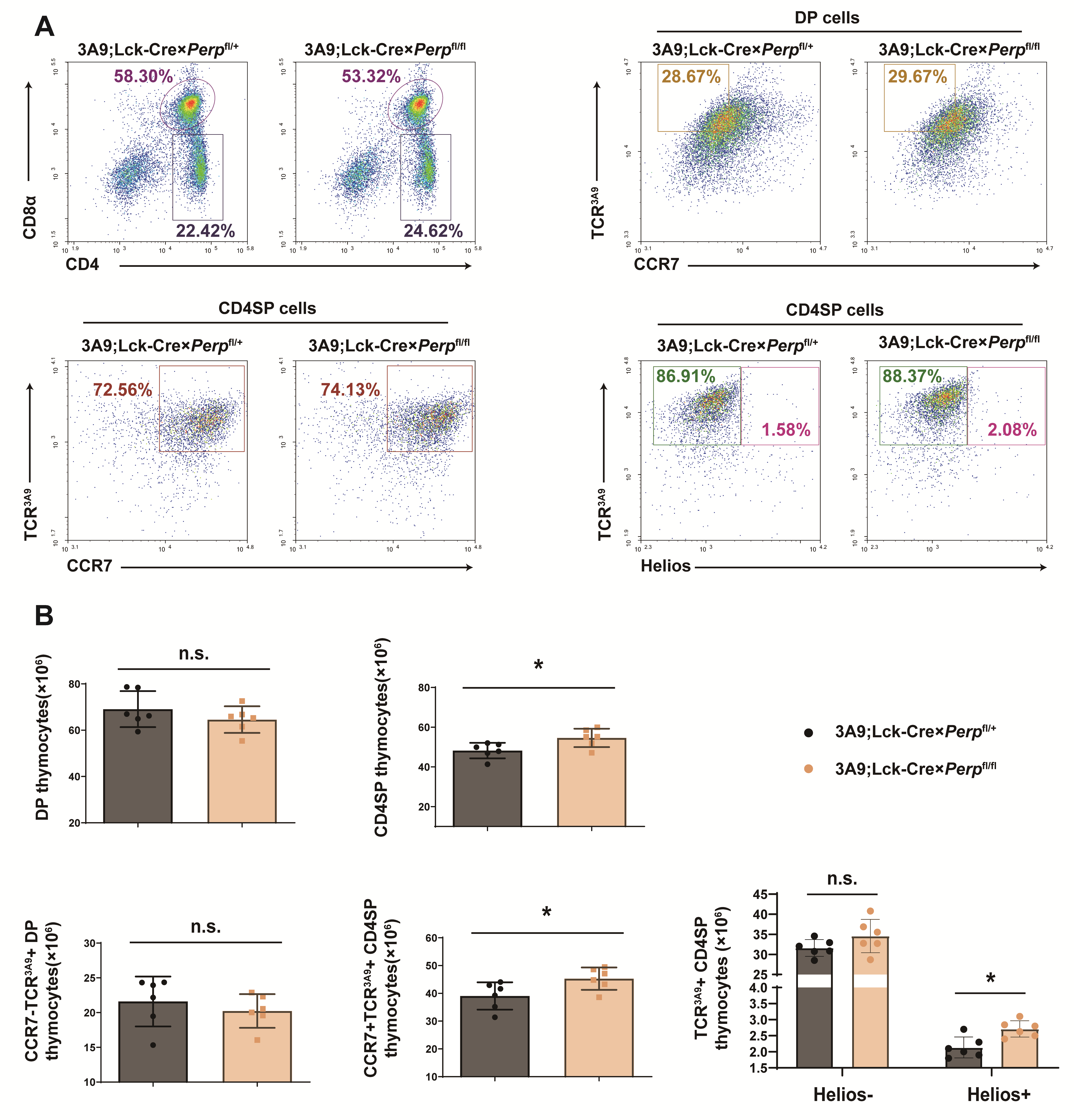


**Fig. S1. *Perp* knock-out does not affect normal positive selection.**

(A) Flow cytometry analysis of thymocytes in 6–8-week-old 3A9 TCR transgenic mice and 3A9;Lck-Cre×*Perp*^fl/fl^ mice according to the expression of CD4, CD8α, CCR7, TCR^3A9^, and Helios. (B) Quantification of DP, CD4SP, CCR7^-^TCR^3A9+^ DP, CCR7^+^TCR^3A9+^ CD4SP, Helios^-^TCR^3A9+^ CD4SP, and Helios^+^TCR^3A9+^ CD4SP thymocytes, analyzed as in A (6 mice per group).


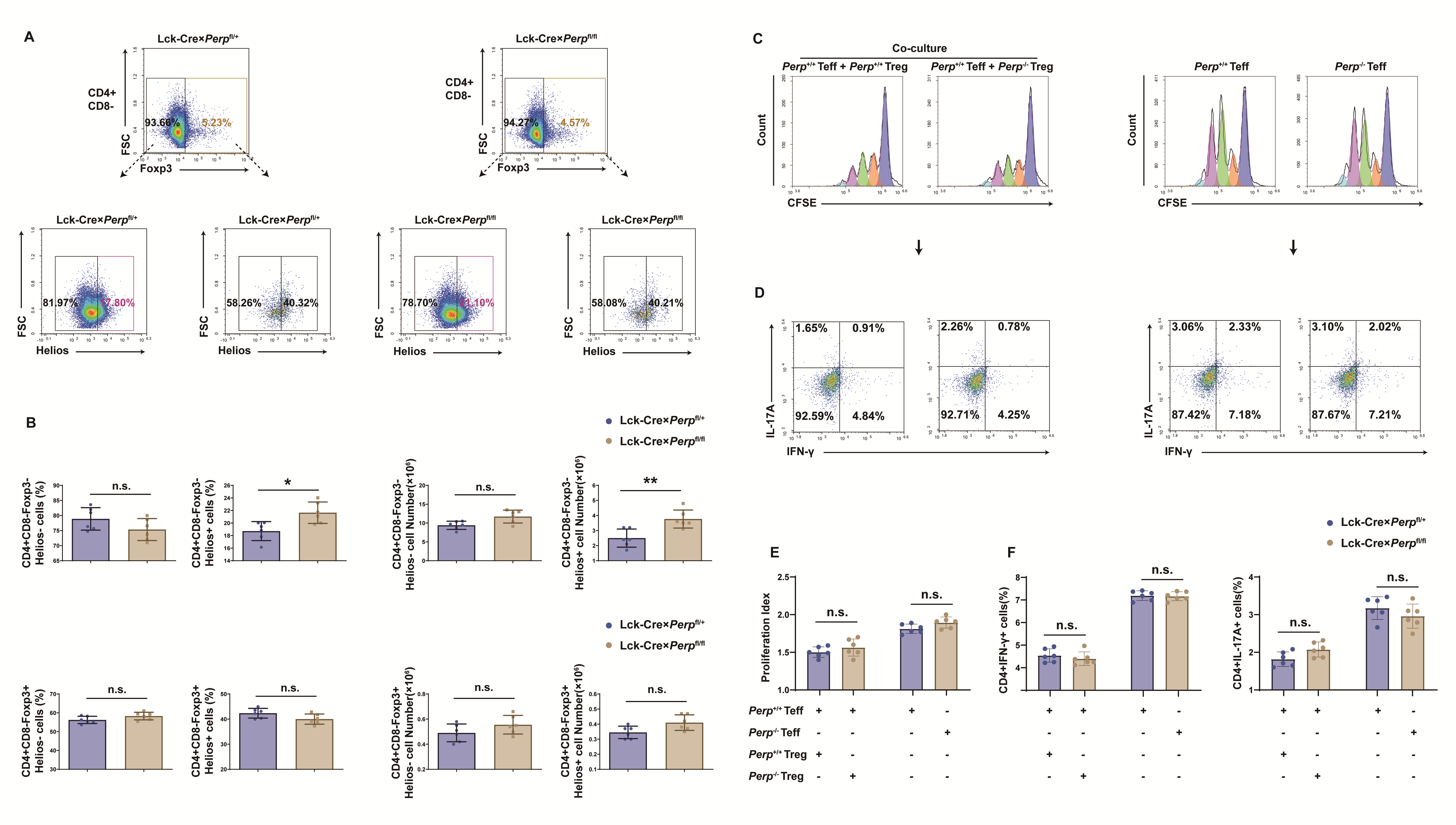


**Fig. S2. *Perp* deficiency does not alter the development of thymic Tregs.**

(A) Flow cytometry analysis of thymocytes in 12-week-old Lck-Cre×*Perp*^fl/+^ mice and Lck-Cre×*Perp*^fl/fl^ mice according to the expression of CD4, CD8α, Foxp3, and Helios. (B) Percentage and number of Foxp3^-^Helios^-^, Foxp3^-^Helios^+^, Foxp3^+^Helios^-^, and Foxp3^+^Helios^+^ CD4SP thymocytes in mice, analyzed as in A (6 mice per group). (C) Flow cytometry analysis of CD4^+^ Teff proliferation (CFSE fluorescence) 3 days after *Perp*^+/+^ or *Perp*^-/-^ Tregs and Teffs were co-cultured in the presence of irradiated bone marrow derived dendritic cells (20 Gy) and the addition of CD3ε/CD28 antibodies (2 μg/mL each). (D) Gating of CD4^+^ T-cells and analysis of intracellular expression of IFNγ and IL-17A under various conditions. (E) Cell proliferation index (from the analysis module in NovoExpress) of CD4^+^ Teffs (6 mice per group). (F) Percentages of CD4^+^IFNγ^+^ T-cells and CD4^+^IL-17A^+^ T-cells, analyzed as in D (6 mice per group).


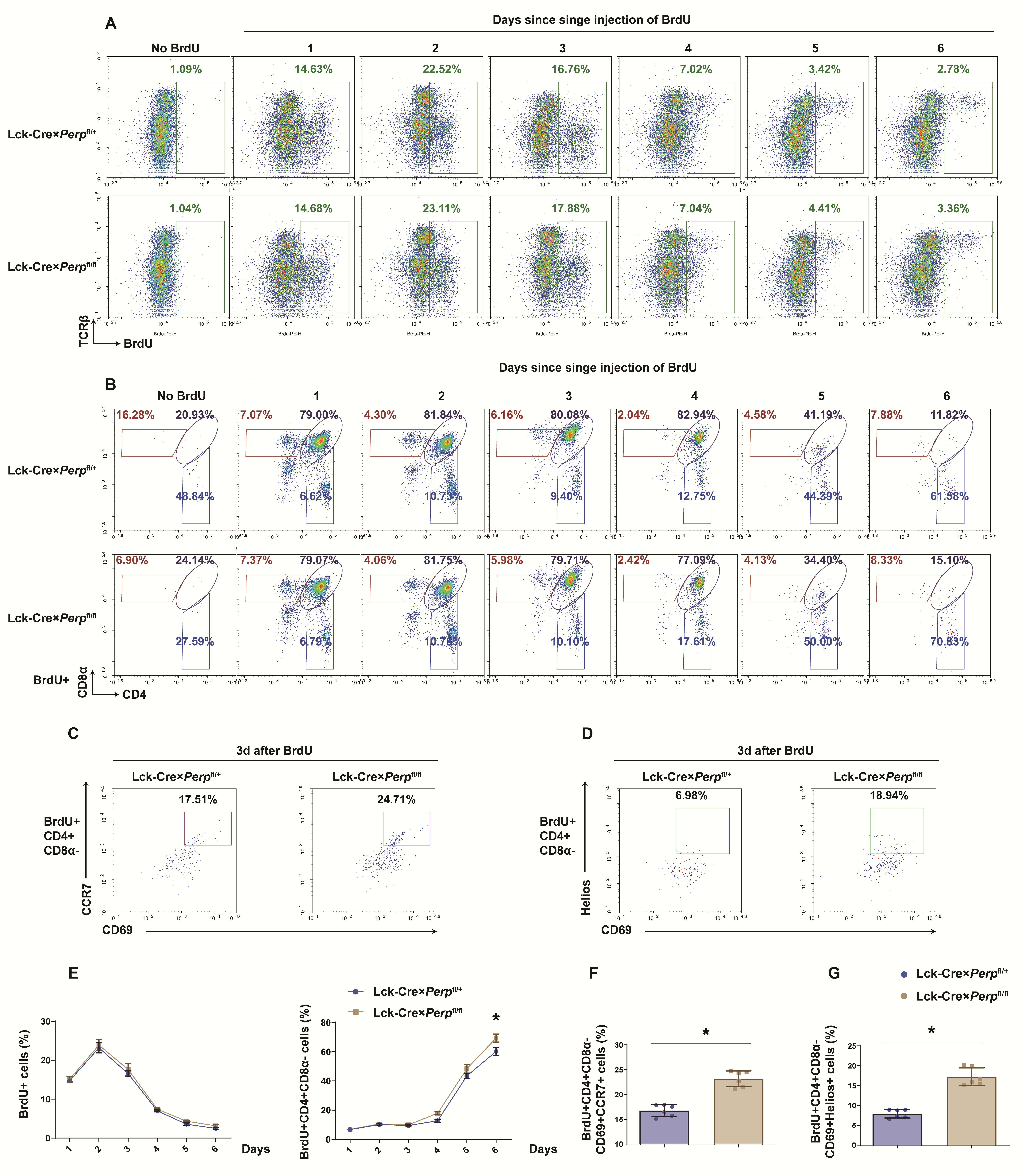


**Fig. S3. Lck-Cre×*Perp*^fl/fl^ mice have normal thymocyte proliferation.**

(A) Flow cytometry analysis of thymocytes from Lck-Cre×*Perp*^fl/+^ mice and Lck-Cre×*Perp*^fl/fl^ mice, which did nor did not receive intraperitoneal BrdU (1 mg) on 1, 2, 3, 4, 5, or 6 days previously, with labeling of TCRβ and BrdU. (B) Flow cytometry analysis with labeling for CD4 and CD8α in BrdU^+^ thymocytes. (C) Flow cytometry analysis of BrdU^+^ CD4SP thymocytes in mice 3 days after BrdU administration, according to the expression of CD69 and CCR7. (D) Flow cytometry analysis of BrdU^+^ CD4SP thymocytes in mice 3 days after BrdU administration, according to the expression of CD69 and Helios markers. (E) Quantification of BrdU^+^ thymocytes in mice, analyzed as in A and B (6 mice per group). (F) Quantification of CD69^+^CCR7^+^BrdU^+^ CD4SP thymocytes in mice 3 days after BrdU administration, analyzed as in C (6 mice per group). (G) Quantification of CD69^+^Helios^+^BrdU^+^ CD4SP thymocytes in mice 3 days after BrdU administration, analyzed as in C (6 mice per group).
